# Annexin A3 as a Marker Protein for Microglia in the Central Nervous System of Rats

**DOI:** 10.1155/2021/5575090

**Published:** 2021-06-10

**Authors:** Zengli Zhang, Zhengyiqi Li, Zhi Ma, Meiling Deng, Manyu Xing, Jing Wu, Shasha Jiang, Qiang Wang, Qulian Guo, Wangyuan Zou

**Affiliations:** ^1^Department of Anesthesiology, Xiangya Hospital, Central South University, Changsha 410008, China; ^2^Department of Anesthesiology, Center for Brain Science, The First Affiliated Hospital of Xi'an Jiaotong University, Xi'an, China; ^3^National Clinical Research Center for Geriatric Disorders, Xiangya Hospital, Central South University, Changsha 410008, China

## Abstract

The parenchymal microglia possess different morphological characteristics in cerebral physiological and pathological conditions; thus, visualizing these cells is useful as a means of further investigating parenchymal microglial function. Annexin A3 (ANXA3) is expressed in microglia, but it is unknown whether it can be used as a marker protein for microglia and its physiological function. Here, we compared the distribution and morphology of parenchymal microglia labeled by ANXA3, cluster of differentiation 11b (CD11b), and ionized calcium-binding adaptor molecule 1 (Iba1) and measured the expression of ANXA3 in nonparenchymal macrophages (meningeal and perivascular macrophages). We also investigated the spatiotemporal expression of ANXA3, CD11b, and Iba1 in vivo and in vitro and the cellular function of ANXA3 in microglia. We demonstrated that ANXA3-positive cells were abundant and evenly distributed throughout the whole brain tissue and spinal cord of adult rats. The morphology and distribution of ANXA3-labeled microglia were quite similar to those labeled by the microglial-specific markers CD11b and Iba1 in the central nervous system (CNS). ANXA3 was expressed in the cytoplasm of microglia, and its expression was significantly increased in activated microglia. ANXA3 was almost undetectable in the nonparenchymal macrophages. Meanwhile, the protein and mRNA expression levels of ANXA3 in different regions of the CNS were different from those of CD11b and Iba1. Moreover, knockdown of ANXA3 inhibited the proliferation and migration of microglia, while overexpression of ANXA3 enhanced these activities. This study confirms that ANXA3 may be a novel marker for parenchymal microglia in the CNS of adult rats and enriches our understanding of ANXA3 from expression patterns to physiological function.

## 1. Introduction

The parenchymal microglia are critical cells that constitute approximately 15%-20% of the total number of glial cells in the central nervous system (CNS) [1]. They are highly motile cells distributed throughout the cerebral tissue and constantly survey tissue homeostasis with micromovements of their distal processes [1, 2]. In addition, microglia are activated by various environmental stimuli caused by brain injury or neurological diseases and are responsible for the clearing of dead tissue and toxic substances to maintain tissue homeostasis [1, 3]. Microglial activation involves morphological changes in which the cells develop shortened cytoplasmic processes and large soma [2]. Thus, detecting the different states of cellular activation by visualizing the cell morphology of parenchymal microglia is vital in the field of microglial research.

Existing visualization techniques are based on surface molecules expressed in resting and activated parenchymal microglial cells. The widely used classical microglial-specific markers are the cluster of differentiation 11b (CD11b) and ionized calcium-binding adaptor molecule 1 (Iba1). They are constitutively expressed by all resting and activated microglial subpopulations, as well as by peripheral macrophages, such as splenic macrophages and Kupffer cells [4–7]. Annexin 3 (ANXA3) belongs to the structurally related annexin protein family, whose members bind to negatively charged phospholipids in the presence of calcium and participate in membrane trafficking, endocytosis, and cytoskeletal modulation [8–10]. The study by Junker et al. [8] showed that ANXA3 is highly expressed in resting and activated microglia in the rat brain following reversible cerebral ischemia. We and other investigators also found that ANXA3 is abundantly expressed in the microglia in the spinal cord of rats and mice [9, 11, 12]. However, whether ANXA3 can be used as a marker protein for microglia and the physiological function of ANXA3 in microglia is mostly unknown.

In the present study, to confirm the possibility of ANXA3 as a novel parenchymal microglial marker for adult rats and to gain insight into its cellular functions, we investigated the immunostaining patterns and expression levels of ANXA3, CD11b, and Iba1 in different regions of the CNS at different developmental time points and the roles of microglial ANXA3 in cell migration and cell proliferation.

## 2. Material and Methods

### 2.1. Animals

Sprague-Dawley (SD) male rats aged 1, 3, 6, 9, and 12 months were obtained from the Experimental Animal Center of Central South University (Changsha, China). The animals were housed in a specific pathogen-free and temperature-controlled facility. The animals were kept under a 12-hour light-dark cycle and were given free access to food and water. All animal experimental procedures were approved by the Ethics Committee for Animal Experimentation of Central South University and followed the Chinese National Institutes of Health Guide for the Care and Use of Laboratory Animals.

### 2.2. Middle Cerebral Artery Occlusion (MCAO)

MCAO was performed as previously described [13]. Briefly, general anesthesia was induced by inhalational isoflurane (2%). Then, the middle cerebral artery was occluded using a nylon monofilament inserted from the left common carotid artery (CCA). The cerebral blood flow was recovered by removing the filament 90 min after MCAO. Immunofluorescence staining was performed 3 days after reperfusion to observe the morphology of microglial cells in the ischemic penumbra.

### 2.3. Embryo Collection

Male and female adult SD rats (3 months) were paired overnight. The next morning was designated embryonic day 0.5 (E0.5) if a vaginal plug was present (7:00 am) at the vaginal opening of female rats. Rat brains of the embryos were collected at E9.5, E11.5, E13.5, E15.5, E17.5, and E19.5.

### 2.4. Cell Cultures

Immortalized murine microglial N9 cells were a gift from Dr. Zhi Ma (Department of Anesthesiology, the First Affiliated Hospital of Xi'an Jiaotong University, China). The N9 cells were cultured in Iscove's modified Dulbecco's medium (IMDM, HyClone) containing 10% fetal bovine serum (FBS, Gibco) and 1% penicillin/streptomycin. The cells were maintained in a 37°C sterile container containing a mixture of 95% atmospheric air and 5% CO_2_. The medium was replaced every 3 days.

### 2.5. Lentiviral Transfection

Up- and downregulation of the ANXA3 protein expression in microglial N9 cells was achieved via lentiviral transfection (LV-ANXA3 and LV-shANXA3, respectively) as previously described [14], Briefly, N9 cells were seeded in 96-well culture plates. The lentivirus (diluted using DMEM) was added to infect the cells for 24 h (multiplicity of infection, MOI = 80). Puromycin (4.5 *μ*g/ml; Sigma, USA) selection was then performed to kill the uninfected cells. The LV-ANXA3, LV-shANXA3 (5′-CGGCCATCCAATCAGATACTT-3′) targeting the mouse ANXA3 sequence (GenBank Accession: NM_013470.2, gene name: annexin A3 or Anxa3), an additional scrambled sequence (LV-NC; 5′-TTCTCCGAACGTGTCACGT-3′), and LV-empty were synthesized by Suzhou GenePharma (China). Briefly, to produce the lentivirus, 70% confluent 293T cells were cotransfected with lentivirus vector and plasmids (PG-P1-VSVG, PG-P2-REV, and PG-P3-RRE) using RNAi-Mate (Suzhou GenePharma, China). The medium was replaced 6 h after transfection. Lentiviral particles were harvested from the supernatant 72 h after the transfection and purified by ultracentrifugation. These particles are hereafter referred to as LV-ANXA3, LV-shANXA3, LV-NC (negative control, a control for LV-shANXA3), and LV-empty (a control for LV-ANXA3). The transfection efficiency of the lentivirus was the number of successfully transfected cells/the number of total cells in the same field of view. Up- and downregulation of ANXA3 protein expression was identified by Western blotting.

### 2.6. Cytoplasmic and Nuclear Protein Extraction

The cytoplasmic and nuclear protein extraction of N9 cells was performed using a Minute™ Cytoplasmic and Nuclear Extraction Kit for Cells (SC-003, Invent Biotechnologies, China) strictly according to the product instructions.

### 2.7. Cell Cycle Analysis

The cell cycle distribution of N9 cells was assessed by flow cytometry as previously described [14]. Briefly, N9 cells were collected, fixed in ice-cold 75% ethanol, and stained with propidium iodide solution (BD Biosciences, China). The DNA content was determined by flow cytometry data using CellQuest Software. For each sample, 10,000 events were counted (FACSCalibur, Becton-Dickinson, USA). The percentage of cells that were in a particular cell cycle stage was calculated using FlowJo software.

### 2.8. EdU Proliferation Assay

The proliferation of the N9 cells was determined using a Cell-Light EdU (5-ethynyl-20-deoxyuridine) Apollo 567 In Vitro Kit (RiboBio, China) according to the product instructions. The percentage of EdU-positive cells was calculated from five random fields in three wells.

### 2.9. Transwell Migration Assays

Cell migration was evaluated using a Transwell chamber with an 8 *μ*m pore filter membrane (Corning, USA) as previously described [15]. Approximately 2 × 10^5^ cells in serum-free medium were seeded in the upper chamber, while 600 *μ*l of IMDM medium supplemented with 20% FBS was added to the lower chamber. Cells were incubated at 37°C in a humidified incubator containing 5% CO_2_ for 24 hours. Cells that migrated into the lower chamber were stained with 10% crystal violet (Sigma, USA) and counted in five random areas under a light microscope.

### 2.10. Wound Healing Assay

A wound healing assay was performed to observe the cell migration of microglia as previously described [16]. Transverse lines were drawn at the back of the 6-well culture plate evenly with a marking pen, and N9 cells were seeded in the plates and allowed to reach 100% confluence. An artificial wound was made using a 10 *μ*l pipette tip (the same pipette tip was used to make scratches for all the wells) across the cell monolayer perpendicular to the lines drawn with the marking pen. Cells were rinsed with PBS and cultured in serum-free medium. Images of the cells were captured at 0 and 24 hours after scratching. The cell migration index was quantified by (D0-D24)/D0, where D0 is the distance from the wound edges at 0 hours, and D24 is the distance from the wound edges at 24 hours.

### 2.11. Immunohistochemical Staining (IHC)

Immunohistochemical staining was performed using a PV-9000 Kit (ZSBiO, China) according to the product instructions [17]. Briefly, brain and spinal cord sections from rats were sliced into 10 *μ*m thick sections (Leica CM1900), which were then subjected to antigen retrieval in a heated citrate buffer (pH 6.0) and incubated with 3% H_2_O_2_ for 10 min at room temperature to block endogenous peroxidase activity. Rabbit anti-ANXA3 antibody (1 : 1000, Sigma, USA) was used as a primary antibody. After the sections were incubated with peroxidase-labeled goat anti-rabbit/mouse IgG antibody, diaminobenzidine (DAB) was used as a chromogen.

### 2.12. Immunofluorescence Staining Assay

Immunofluorescence staining was performed on either frozen coronal sections of rats or N9 cells plated on cover slips as previously described [18]. The following primary antibodies were used: mouse anti-CD206 antibody (GB13438, 1 : 200, Servicebio, China), mouse anti-CD11b antibody (ab1211, 1 : 200, Abcam, England), rabbit anti-ANXA3 antibody (HPA013398, 1 : 200, Sigma, USA), and goat anti-Iba1 antibody (ab5076, 1 : 200, Abcam, England). Then, the samples were incubated with mixtures of Alexa-488- (green, Invitrogen), Alexa-594- (red, Santa Cruz), and Alexa-647- (red, Invitrogen) conjugated donkey anti-goat, anti-rabbit, and anti-mouse secondary antibodies. Finally, the sections were viewed under a fluorescence microscope (Olympus BX51, Japan) and photographed.

### 2.13. Western Blotting

The ANXA3, CD11b, and Iba1 protein expression levels were measured by Western blotting as described previously [19]. The following primary antibodies were used: rabbit anti-CD11b antibody (ab133357, 1 : 1000, Abcam, England), rabbit anti-ANXA3 antibody (HPA013398, 1 : 1000, Sigma, USA), rabbit anti-Iba1 (1 : 500, Wako, Japan), and rabbit anti-tubulin antibody (1 : 1000, Cell Signaling Technology, USA). The membranes were then incubated with an HRP-conjugated anti-rabbit or anti-mouse secondary antibody (Thermo Scientific) for two hours. Protein bands were visualized using a LI-COR Odyssey System (LI-COR Biotechnology).

### 2.14. Quantitative (Real-Time) Polymerase Chain Reaction (Real-Time PCR)

The ANXA3 mRNA expression levels in the olfactory bulb, cortex, hippocampus, thalamus, cerebellum, and spinal cord collected from SD rats aged 1, 3, 6, 9, and 12 months were measured by real-time PCR as previously described. Total RNA was isolated using a kit (ER501-01, Transgen, China) and reverse transcribed in a 20 *μ*l reaction at 42°C for 15 min followed by 85°C for 5 sec using SuperMix (M10111, Transgen, China). Real-time PCR was performed according to the manufacturer's manual of the qPCR Kit (AQ141, Transgen, China). The reaction was performed at 94°C for 30 sec followed by 40 cycles of 94°C for 5 sec, 60°C for 15 sec, and 72°C for 19 sec on the ViiA7 Real-Time PCR Detection System (2720 Thermal Cycler, Applied Biosystems). The mRNA primer sequences used are listed in Table 1. The relative mRNA expression was analyzed using the formula 2^-(Ct target gene-Ct reference gene)^.

### 2.15. Statistical Analysis

Statistical tests were performed using PRISM v7.0 software (GraphPad). Data were collected by an independent investigator who was blinded to the groups. Comparisons between two groups were performed using Student's *t*-tests whereas comparisons among more than two groups were performed using one-way ANOVA with Tukey's posttest. All values are presented as the means ± SD. Values of *p* < 0.05 were considered statistically significant.

## 3. Results

### 3.1. Colocalization of ANXA3, CD11b, and Iba1 within Microglia in Different Regions

**Figure 1:**
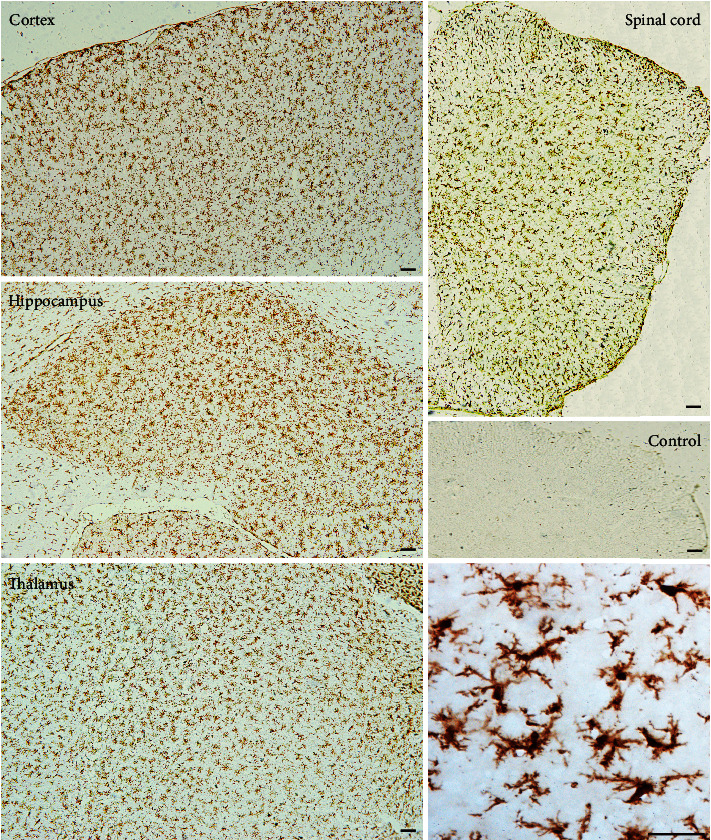
Overall pattern of ANXA3 immunoreactivity in the CNS of rats. Representative immunohistochemical images showing the immunoreactivity of ANXA3 in the cortex, hippocampus, thalamus, and spinal cord and the negative control. The negative control was the cortex tissue that was performed with IHC without a primary antibody. The image in the lower right corner shows the morphology of ANXA3-positive cells in the thalamus at a higher magnification. Scale bar = 100 *μ*m.

**Figure 2:**
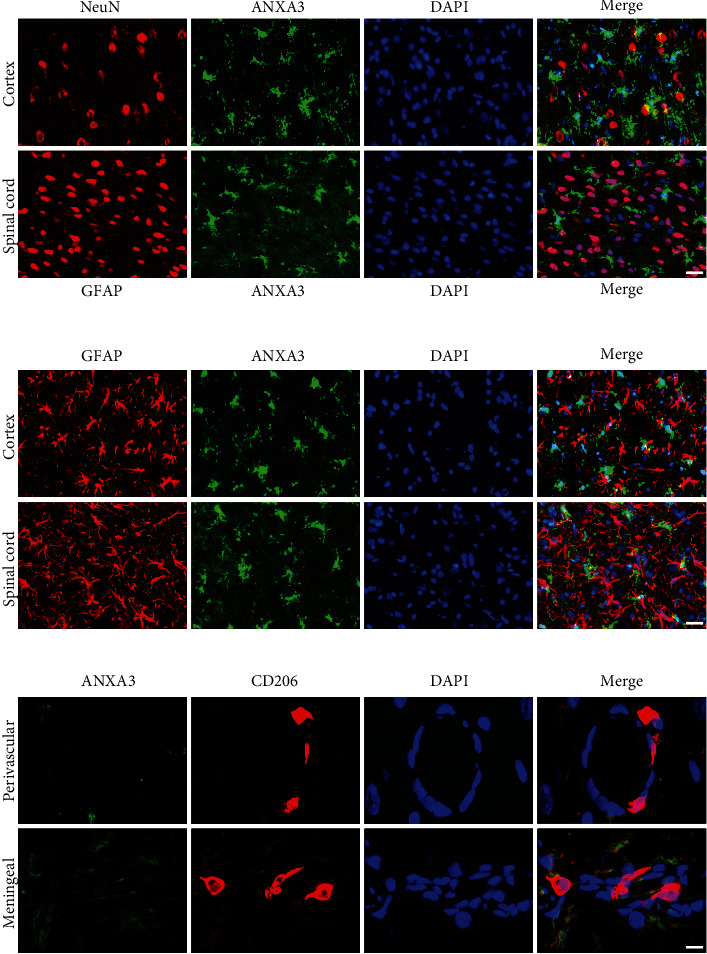
ANXA3 immunoreactivity in neurons, astrocytes, and nonparenchymal macrophages. (a, b) Representative immunofluorescence images showing the expression of ANXA3 in neurons (stained with NeuN) and astrocytes (stained with GFAP) from the cortex and spinal cord. Scale bar = 20 *μ*m. (c) Representative immunofluorescence images showing the expression of ANXA3 in macrophages labeled with CD206 in the perivascular and meningeal. Scale bar = 50 *μ*m.

**Figure 3:**
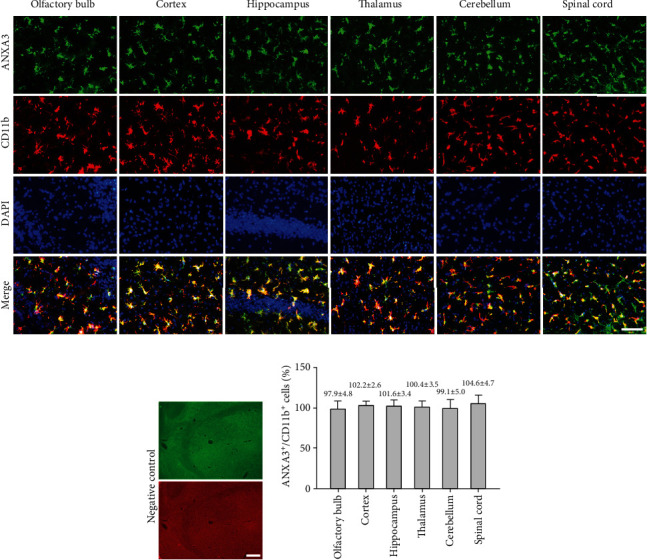
Colocalization of ANXA3 and CD11b in different regions of the CNS. (a) Representative immunofluorescence images of microglia labeled by ANXA3 and CD11b in the olfactory bulb, cortex, hippocampus, thalamus, cerebellum, and spinal cord of adult rats (3 months). Scale bar = 40 *μ*m. (b) Images of the negative control (without primary antibody). Scale bar = 100 *μ*m. (c) Quantitative analysis of ANXA3^+^/CD11b^+^ microglial cells in different regions of the CNS. The ANXA3^+^/CD11b^+^ are the number of ANXA3-positive cells/the number of CD11b-positive cells in the same randomly selected field of view. The data are presented as the means ± SD and were analyzed by one-way ANOVA with Tukey's post hoc test. *n* = 5 per group. Cells in five randomly selected fields were counted.

**Figure 4:**
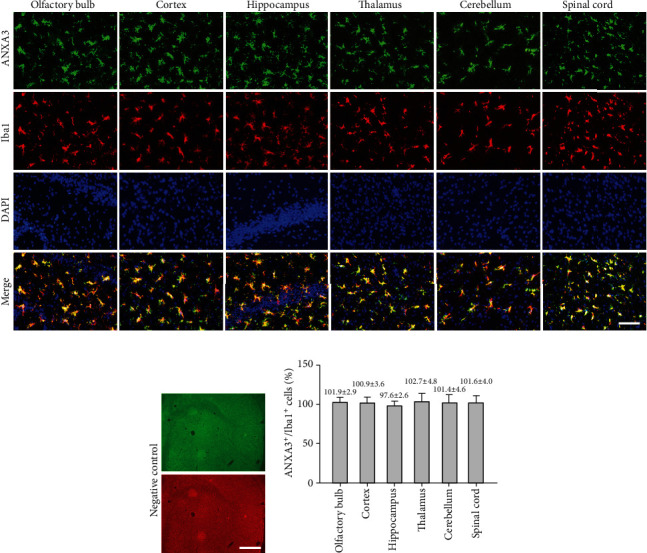
Colocalization of ANXA3 and Iba1 in different regions of the CNS. (a) Representative immunofluorescence images of microglia labeled by ANXA3 and Iba1 in the olfactory bulb, cortex, hippocampus, thalamus, cerebellum, and spinal cord of adult rats (3 months). Scale bar = 40 *μ*m. (b) Images of the negative control (without primary antibody). Scale bar = 100 *μ*m. (c) Quantitative analysis of ANXA3^+^/Iba1^+^ microglial cells in different regions of the CNS. The data are presented as the means ± SD and were analyzed by one-way ANOVA with Tukey's post hoc test. *n* = 5 per group. Cells in five randomly selected fields were counted.

Brain sections that were immunohistochemically processed for light microscopy showed that ANXA3 immunoreactive cells were abundant and evenly distributed in the cortex, hippocampus, thalamus, and spinal cord of the adult rats (3 months) (Figure 1). When viewed at a higher magnification, the ANXA3-positive cells presented a radial shape characterized by small soma, long processes, and multiple branches (Figure 1), particularly similar to the morphology of glial cells. To confirm the phenotype of ANXA3-positive cells, we performed double immunofluorescence staining with NeuN, the neuronal nuclei-specific marker, and GFAP, the astrocyte marker, in the cortex and spinal cord of adult rats (3 months). Immunostaining showed that ANXA3 was almost undetectable in astrocytes labeled with GFAP and neurons labeled with NeuN in the cortex and spinal cord (Figures 2(a) and 2(b)). As brain macrophages including microglia can be distinguished into parenchymal microglia and CD206^+^ CNS-associated macrophages (CAMs), namely, the nonparenchymal macrophages (perivascular macrophages, meningeal macrophages) [20], we detected the expression of AXNA3 in perivascular macrophages and meningeal macrophages using immunofluorescence staining (Figure 2(c)). Immunostaining showed that ANXA3 was almost undetectable in macrophages labeled with CD206 in the perivascular and meningeal (Figure 2(c)). We then detected the colocalization of ANXA3 with the microglial-specific markers CD11b and Iba1 throughout the brain and spinal cord of adult rats. The results indicate that ANXA3 and CD11b presented nearly complete colocalization in the microglia from the olfactory bulb, cortex, hippocampus, thalamus, cerebellum, and spinal cord of the adult rats (Figure 3(a)).  Figure 3(b) shows images of the negative control (without primary antibody). Meanwhile, immunostaining showed that ANXA3 was nearly colocalized with Iba1 throughout the cerebrum, cerebellum, and spinal cord of the adult rats (Figure 4(a)).  Figure 4(b) shows images of the negative control (without primary antibody). No significant differences in the ANXA3^+^/CD11b^+^ cells (Figure 3(c)) and ANXA3^+^/Iba1^+^ cells (Figure 4(c)) were observed among the different regions.

### 3.2. The Morphologies of Resting and Activated Microglia Labeled by ANXA3

**Figure 5:**
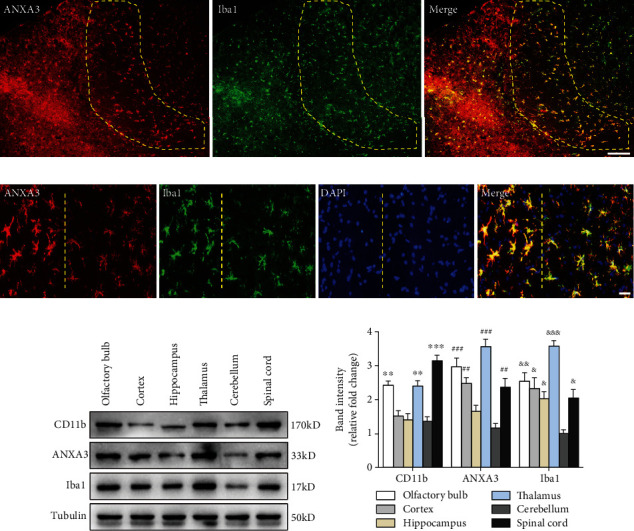
Morphologies of microglia labeled by ANXA3 and ANXA3 protein expression levels in different regions. (a) Low-magnification micrographs showing microglial cells in the ischemic penumbra 3 days after reperfusion. The yellow-dotted ring indicates the ischemic penumbra area between the infarct area and the normal area of the brain tissue. Scale bars = 200 *μ*m. (b) Representative immunofluorescence images showing the morphology of activated microglia in the ischemic penumbra (on the left) and resting microglia in the healthy area (on the right) labeled by ANXA3 and Iba1 at high magnification. Scale bars = 50 *μ*m. *n* = 5 per group. (c, d) The expression levels of ANXA3, CD11b, and Iba1 proteins in the olfactory bulb, cortex, hippocampus, thalamus, cerebellum, and spinal cord of adult rats (3 months) were determined by Western blotting analysis. The panel shows protein bands corresponding to ANXA3, CD11b, Iba1, and tubulin. The histogram shows the results of the densitometric analysis. The data are expressed as the means ± SD and were analyzed by one-way ANOVA with Tukey's post hoc test. ^∗∗^*p* < 0.01 and ^∗∗∗^*p* < 0.001 compared with the cerebellum group. ^##^*p* < 0.01 and ^###^*p* < 0.001 compared with the cerebellum group. ^&^*p* < 0.05, ^&&^*p* < 0.01, and ^&&&^*p* < 0.001 compared with the cerebellum group. *n* = 6 per group.

As shown in Figure 5(a), the ischemic penumbra was the area between the infarct core and the unaffected (normal) tissue. At a higher magnification, the resting microglia labeled with ANXA3 in the unaffected tissue had a small perinuclear cytoplasm with long, thick processes extending in multiple directions, while the activated microglia labeled by ANXA3 in the penumbra displayed larger soma and shorter, coarser cytoplasmic processes, which were particularly similar to those of the microglial cells labeled by Iba1 (Figure 5(b)).

### 3.3. Protein Expression Levels of ANXA3, CD11b, and Iba1 in Different Regions

We performed Western blotting to measure the protein expression levels of ANXA3, CD11b, and Iba1 in the olfactory bulb, cortex, hippocampus, thalamus, cerebellum, and spinal cord of the adult rats (Figures 5(c) and 5(d)). The levels of ANXA3 protein expression in the olfactory bulb, cortex, thalamus, and spinal cord were much higher than those in the cerebellum, while no significant difference was observed between the hippocampus and cerebellum. The levels of CD11b protein expression in the olfactory bulb, thalamus, and spinal cord were much higher than those in the cerebellum, and no significant differences were observed among the cortex, hippocampus, and cerebellum. The level of Iba1 protein expression in the cerebellum was significantly lower than those in the other regions, and no significant differences were observed among the olfactory bulb, cortex, hippocampus, and spinal cord.

### 3.4. Spatiotemporal Expression of ANXA3 in the CNS of Rats

**Figure 6:**
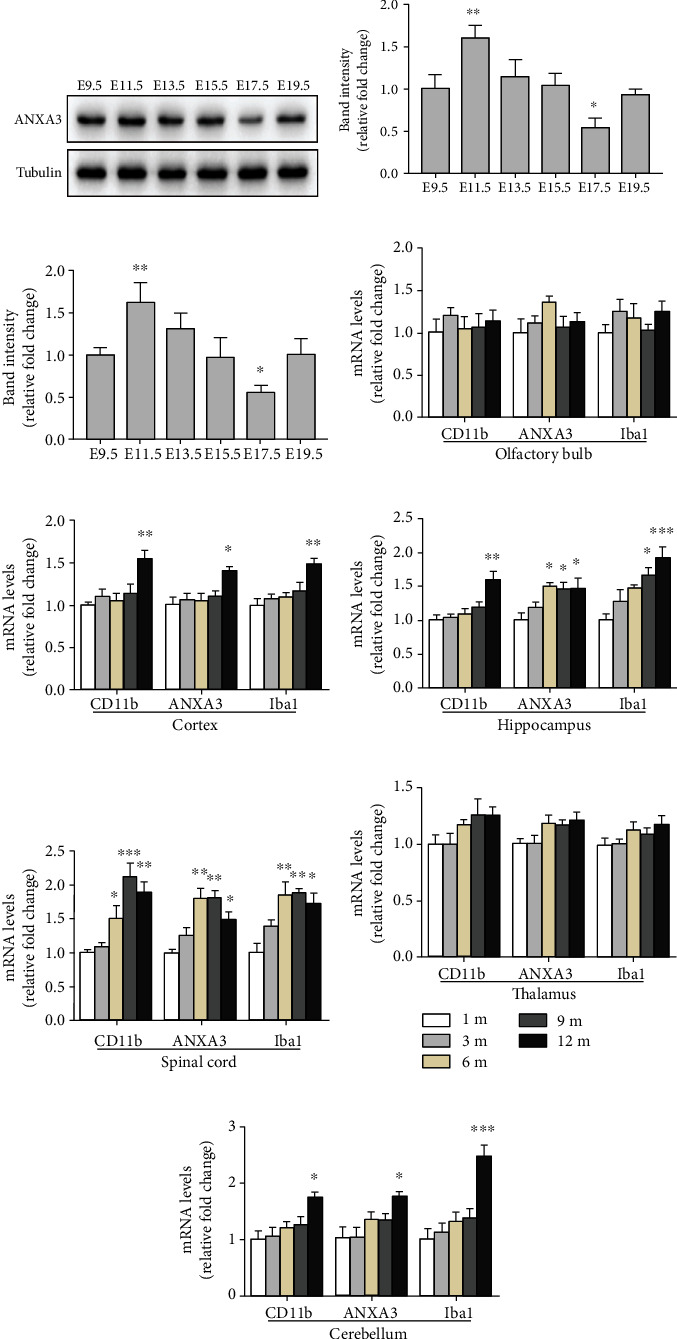
Spatiotemporal expression of ANXA3 protein and mRNA. (a, b) The expression levels of ANXA3 protein in rat embryos from E9.5 to E19.5 were determined by Western blotting analysis. The panel shows the protein bands corresponding to ANXA3 and tubulin. The histogram indicates the quantification of the densitometric analysis. The data are expressed as the means ± SD and were analyzed by one-way ANOVA with Tukey's post hoc test. ^∗^*p* < 0.05 and ^∗∗^*p* < 0.01 compared with the E9.5 group. *n* = 6 per group. (c) Real-time PCR analysis of ANXA3 mRNA expression in rat embryos from E9.5 to E19.5. The data are expressed as the means ± SD and were analyzed by one-way ANOVA with Tukey's post hoc test. ^∗^*p* < 0.05 and ^∗∗^*p* < 0.01 compared with the E9.5 group. *n* = 6 per group. (d–i) The levels of ANXA3 mRNA expression in the olfactory bulb, cortex, hippocampus, thalamus, cerebellum, and spinal cord of rats aged 1, 3, 6, 9, and 12 months were determined by qPCR analysis. The data are expressed as the means ± SD and were analyzed by one-way ANOVA with Tukey's post hoc test. ^∗^*p* < 0.05, ^∗∗^*p* < 0.01, and ^∗∗∗^*p* < 0.001 compared with the 1 m group. *n* = 6 per group. 1 m: 1 month; 3 m: 3 months; 6 m: 6 months; 9 m: 9 months; 12 m: 12 months.

ANXA3 protein and mRNA expression levels were examined to determine the overall trend of the ANXA3 expression in embryos from E9.5 to E19.5 (Figures 6(a)–6(c)). The levels of the ANXA3 protein and mRNA expression were highest at E11.5 and lowest at E17.5 during brain development. No significant differences were observed among the E9.5, E13.5, E15.5, and E19.5 groups. We also measured the mRNA expression levels of ANXA3, CD11b, and Iba1 in the olfactory bulb, cortex, hippocampus, thalamus, cerebellum, and spinal cord of rats aged 1, 3, 6, 9, and 12 months (Figures 6(d)–6(i)). In the olfactory bulb and thalamus, the mRNA expression levels of ANXA3, CD11b, and Iba1 showed no significant changes in rats aged 1, 3, 6, 9, and 12 months (Figures 6(d) and 6(g)); however, mRNA expression levels of all three genes were significantly increased in the cortex and cerebellum of rats aged 12 months compared with rats aged 1 month (Figures 6(e) and 6(h)). In the hippocampus and spinal cord, the mRNA expression levels of ANXA3 and Iba1 were significantly increased starting at 6 months and gradually increased with age (Figures 6(f) and 6(i)). The mRNA expression levels of CD11b in the hippocampus were significantly increased in rats aged 12 months, and the mRNA levels of CD11b in the spinal cord were significantly increased from 6 months and gradually increased with age (Figures 6(f) and 6(i)).

### 3.5. Subcellular Localization of ANXA3 in Microglia

**Figure 7:**
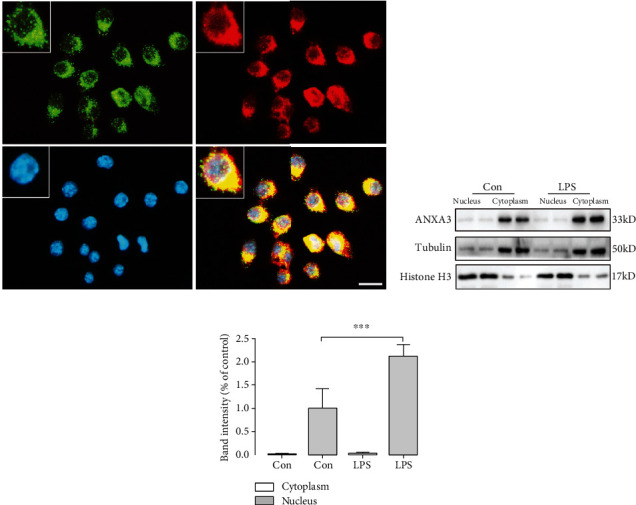
Subcellular localization of ANXA3 in resting and activated N9 microglial cells and lentiviral transfection of N9 cells. (a) Representative immunofluorescence images showing ANXA3 expression in microglial cells. N9 cells were also stained for CD11b expression. The inset shows the microglia at a higher magnification. Scale bar = 40 *μ*m. (b, c) Western blotting analysis of ANXA3 protein expression levels in the cytoplasm and nuclei of resting (Con) and LPS-induced activated microglia (500 ng/ml LPS treatment for 24 hours). The panel shows the ANXA3, tubulin, and histone H3 protein bands, and the histogram reflects the results of the densitometric analysis. The data are expressed as the means ± SD and were analyzed by Student's *t*-tests. ^∗∗∗^*p* < 0.001 compared with the control group. The data were pooled from six independent experiments.

We then investigated the subcellular localization of ANXA3 in resting and activated microglial N9 cells (Figures 7(a)–7(c)). Immunostaining showed that ANXA3 was expressed in N9 cells (Figure 7(a)), with higher magnifications showing that ANXA3 immunoreactivity was mainly present in the cytoplasm (Figure 7(a)). The N9 cells were treated with 500 ng/ml lipopolysaccharide (LPS) for 24 hours to achieve functional activation [21]. ANXA3 protein expression levels in the cytoplasmic and nuclear protein extractions of the resting (Con) and LPS-induced activated N9 cells were detected by Western blotting analysis (Figures 7(b) and 7(c)). The ANXA3 protein was detectable in the cytoplasm of both resting (Con) and LPS-induced activated N9 cells and was almost undetectable in the nuclei of N9 cells (Figures 7(b) and 7(c)). In addition, there was a significant increase in the ANXA3 protein expression in the cytoplasm of LPS-induced activated N9 cells compared with the control resting N9 cells (Figures 7(b) and 7(c)).

### 3.6. ANXA3 Participated in the Proliferation of Microglia

**Figure 8:**
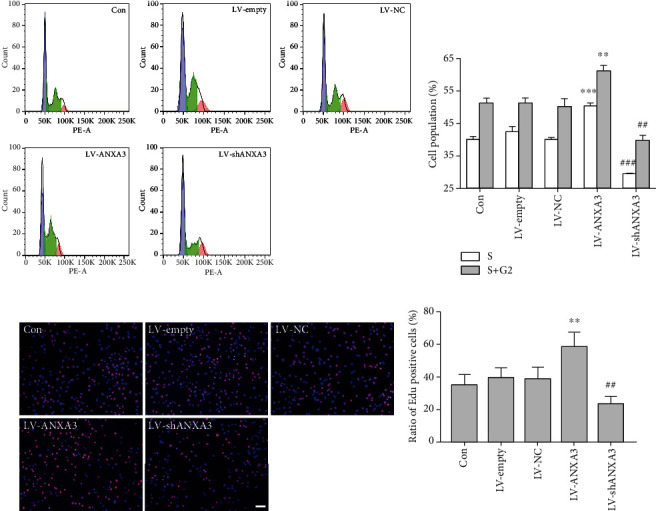
The proliferation of microglial N9 cells. (a, b) Flow cytometry analysis showing the proliferation of the Con, LV-empty, LV-NC, LV-ANXA3, and LV-shANXA3-treated N9 microglial cells. MultiCycle AV software was used to perform the fit analysis of the DNA cell cycle and to calculate the percentages of cells in each phase. The blue peak, the green arc-shaped hatched portion in the middle, and the red peak represent the G1, S, and G2 phases of the cell cycle, respectively. The data are expressed as the means ± SD and were analyzed by one-way ANOVA with Tukey's post hoc test. ^∗∗^*p* < 0.01 and ^∗∗∗^*p* < 0.001 compared with the LV-empty group. ^##^*p* < 0.01 and ^###^*p* < 0.001 compared with the LV-NC group. The data were pooled from five independent experiments. (c, d) The EdU incorporation assay showing the proliferation of the Con, LV-empty, LV-NC, LV-ANXA3, and LV-shANXA3-treated N9 microglial cells. The cell nuclei were stained with DAPI. EdU-positive cells are indicated in pink. The data are expressed as the means ± SD and were analyzed by one-way ANOVA with Tukey's post hoc test. ^∗∗^*p* < 0.01 compared with the LV-empty group. ^##^*p* < 0.01 compared with the LV-NC group. The data were pooled from five independent experiments. Scale bar = 20 *μ*m. DAPI: 4′,6-diamidino-2-phenylindole; EdU: 5-ethynyl-2′-deoxyuridine.

To gain insight into the cellular function of ANXA3 in microglia, overexpression and knockdown of ANXA3 in N9 microglial cells were performed via lentiviral transfection of LV-ANXA3 and ANXA3-targeting shRNA (LV-shANXA3), respectively, and evaluated using Western blotting (Fig. S1). The transfection efficiencies of LV-empty, LV-NC, LV-ANXA3, and LV-shANXA3 were more than 95% (Fig. S1A-S1B). The ANXA3 protein expression was significantly increased in the LV-ANXA3 group compared with the LV-empty group and was significantly decreased in the LV-shANXA3 group compared with the LV-NC group (Fig. S1C-S1D). Next, we measured the proliferative capacity of the overexpressing and knockdown N9 cells using both flow cytometry analysis and EdU incorporation assays. Overexpression of ANXA3 in the LV-ANXA3-treated N9 cells significantly increased the percentages of cells that were in the S phase and S+G2 phase compared to the LV-empty-treated N9 cells, and knockdown of ANXA3 in the LV-shANXA3-treated N9 cells significantly decreased the percentage of cells in the S phase and S+G2 phase compared to the LV-NC-treated N9 cells (Figures 8(a) and 8(b)). The EdU incorporation assay results were consistent with those of the flow cytometry analysis: overexpression of ANXA3 in LV-ANXA3-treated N9 cells significantly increased the percentage of EdU-positive cells compared with LV-NC-treated N9 cells, and knockdown of ANXA3 in LV-shANXA3-treated N9 cells significantly decreased the percentage of EdU-positive cells compared with LV-NC-treated N9 cells (Figures 8(c) and 8(d)).

### 3.7. ANXA3 Participated in the Migration of Microglia

**Figure 9:**
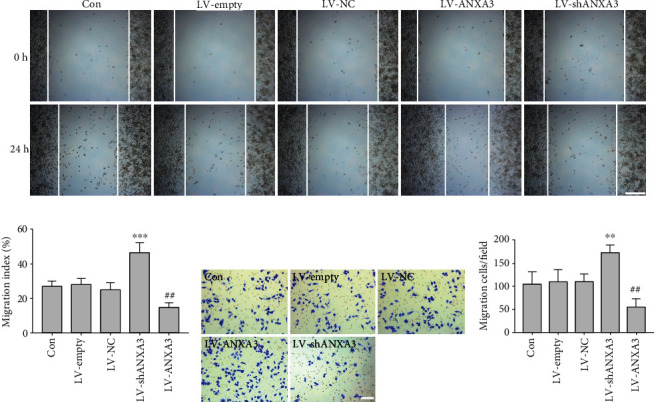
The migration of microglial N9 cells. (a, b) Evaluation of cell migration using a wound healing assay; monolayers were viewed on an inverted microscope. The histogram shows the quantitative analysis of the cell migration index. The data are expressed as the means ± SD and were analyzed by one-way ANOVA with Tukey's post hoc test. ^∗∗∗^*p* < 0.001 compared with the LV-empty group. ^##^*p* < 0.01 compared with the LV-NC group. The data were pooled from five independent experiments. (c, d) Evaluation of cell migration using the Transwell assay; migratory cells were viewed under an inverted microscope. The histogram shows the quantitative analysis of migrating cells. The data are expressed as the means ± SD and were analyzed by one-way ANOVA with Tukey's post hoc test. ^∗∗^*p* < 0.01 compared with the LV-empty group. ^##^*p* < 0.01 compared with the LV-NC group. The data were pooled from five independent experiments.

Wound healing and Transwell assays were performed to determine the migration of microglia. The wound healing assay showed that overexpression of ANXA3 in LV-ANXA3-treated N9 cells significantly increased the migration index of microglia compared with LV-empty-treated N9 cells, and knockdown of ANXA3 in LV-shANXA3-treated N9 cells significantly decreased the migration index of microglia compared with LV-NC-treated N9 cells (Figures 9(a) and 9(b)). The Transwell assay showed that overexpression of ANXA3 in LV-ANXA3-treated N9 cells significantly increased the number of migrating cells compared with LV-empty-treated N9 cells, and knockdown of ANXA3 in LV-shANXA3-treated N9 cells significantly decreased the number of migrating cells compared with LV-NC-treated N9 cells (Figures 9(c) and 9(d)).

## 4. Discussion

ANXA3 is a Ca^2+^-dependent phospholipid-binding protein whose immunostaining patterns and physiological function in the CNS are mostly unknown. In the current study, we demonstrated that ANXA3-positive cells were abundantly and evenly distributed throughout the whole brain tissue and spinal cord of adult rats. The morphology and distribution of ANXA3-positive microglia were quite similar to those of CD11b- and Iba1-positive microglia in the CNS. ANXA3 expression was localized to the cytoplasm of microglia and was increased in activated microglia. Knockdown of ANXA3 inhibited the proliferation and migration of microglia, while overexpression promoted microglial proliferation and migration. The study confirms that ANXA3 may be a novel marker for the parenchymal microglia and can enrich our understanding of ANXA3 in the CNS from expression patterns to physiological functions.

CD11b and Iba1 are molecules used as markers to identify the parenchymal microglial cells in the CNS [4, 5, 22]. The parenchymal microglia labeled by CD11b and Iba1 present a relatively uniform distribution, with the exception of higher relative numbers in the olfactory telencephalon, dentate gyrus of the hippocampus, substantia nigra, and portions of the basal ganglia [3]. Using immunohistochemistry and immunofluorescence, we demonstrated that ANXA3-positive microglia were abundantly and evenly distributed throughout the CNS. Moreover, we found that ANXA3 and CD11b/Iba1 presented nearly complete colocalization in microglia throughout the whole brain tissue and the spinal cord of adult rats. Consistent with our results, a study from Smithson and Kawaja demonstrated that ANXA3-immunopositive cells are evenly distributed throughout the olfactory bulb of adult rats [22]. Microglia are known to undergo morphological transformation in response to various stimuli. Functional changes in microglia are often accompanied by morphological changes to cells with larger soma and shorter, coarser cytoplasmic processes displaying a bushy appearance; these changes can possibly progress to a full amoeboid morphology [3]. In this study, the ANXA3-positive activated microglia in the penumbra presented larger soma and shorter, coarser cytoplasmic processes, particularly similar to Iba1-positive glial cells. The morphology and distribution of ANXA3-positive microglia were quite similar to those of CD11b- and Iba1-positive microglia in the CNS, which appear to identify all the parenchymal microglia; thus, ANXA3 may be a novel marker for the parenchymal microglia. In addition, we found that ANXA3 was nearly undetectable in the perivascular macrophages and meningeal macrophages. Furthermore, we showed that ANXA3 protein expression significantly increased in the cytoplasm of LPS-induced activated microglial cells compared with resting microglia. ANXA3 has been recently reported to be highly upregulated in the postischemic rat brain [8], and ANXA3 knockdown inhibits the nuclear factor-kappa B (NF-*κ*B) pathway by upregulating I*κ*B*α* [23]. These findings indicate that ANXA3 may play a role in the process of microglia activation or microglia-mediated inflammation.

While characteristic features of the microglia structure and function have been identified as they relate to brain development and aging, there are limited experimental data about the changes in the expression of microglial markers across the lifespan of higher organisms. We found that the ANXA3 protein and mRNA expression levels were highest at E11.5 and lowest at E17.5 during the brain development of rats. Microglia originate from progenitor cells in the yolk sac at approximately embryonic day 7.5 (E7.5) and migrate into the rudimentary brain at approximately E9.5 [24]. ANXA3 protein and mRNA were detectable at E9.5 and lasted until E19.5, indicating that ANXA3 may participate in brain development at the embryonic stage. Studies by Dalmau et al. demonstrate that there is a low proportion of microglia undergoing apoptosis during brain development [3, 25]. The decreased expression of ANXA3 at E17.5 may either be due to microglial apoptosis or simply reflect the dramatic changes in volume expansion of the CNS during development. Future investigations will be essential to determine the functional significance of the temporal differential expression of ANXA3 during brain development. Aging is associated with an increase in microglial activation [3, 26]. In both rat and human brains, observations of altered microglia morphology have been observed and are associated with a more reactive/activated phenotype as a function of aging [3, 27–29]. Markers normally present in activated microglia, including MHC II antigens, CD11b, CD14, and pattern recognition receptors, are also elevated in aged microglia [3, 30–34]. In the olfactory bulb and thalamus, the mRNA expression levels of ANXA3, CD11b, and Iba1 showed no changes in rats aged 1, 3, 6, 9, and 12 months; however, in the cortex and cerebellum, the mRNA expression levels of ANXA3, CD11b, and Iba1 were increased in rats aged 12 months. In the hippocampus and spinal cord, the mRNA expression levels of ANXA3, CD11b, and Iba1 gradually increased with age. Differences in the expression levels of ANXA3, CD11b, and Iba1 throughout the brain and spinal cord are suggested to be associated with the microenvironment as well as with functional differences, including receptor expression patterns [35] and the expression of cell surface antigens [36]. In addition, one study has shown that in the hippocampus of mice, ANXA3 is undetectably low at the age of 3 days, significantly increases from 3 days to 3 weeks, and remains high at 3 months [37]. The results from our research and other investigators indicate that ANXA3 plays an important functional role in the CNS across the lifespan of higher organisms.

We also detected the expression levels of ANXA3, CD11b, and Iba1 proteins and mRNA in the olfactory bulb, cortex, hippocampus, thalamus, cerebellum, and spinal cord of adult rats. The expression levels of ANXA3, CD11b, and Iba1 are varied in different regions, indicating the heterogeneity of the marker expression patterns. Expression differences in these cell surface antigens throughout the brain are suggested to be associated with differences in the localization of microglia [3]. In addition, as microglial density has been shown to differ between males and females across stages of the lifespan and throughout brain regions under steady-state conditions and in response to chronic stress [38], future investigations are required to determine whether ANXA3 expression differs between males and females.

Our group originally found that ANXA3 is greatly upregulated in the spinal cord following chronic constriction injury- (CCI-) induced neuropathic pain [9]. Moreover, ANXA3 downregulation alleviates CCI-induced mechanical allodynia and thermal hyperalgesia, indicating that ANXA3 plays a crucial role in neuropathic pain. In addition, studies have shown that overexpression of ANXA3 promotes tumor proliferation in lung, liver, and ovarian carcinomas [23, 39]. ANXA3 plays pivotal roles in promoting cancer and stem cell-like features in CD133^+^ liver cancer stem cells (CSCs) through a dysregulated JNK pathway [40], and overexpression of ANXA3 suppresses apoptosis and promotes autophagic responses via PKCd/p38 MAPK signaling in sorafenib-resistant hepatocellular carcinoma cells (HCCs) [41]. Moreover, downregulation of ANXA3 promotes the repair and healing of myocardial tissues by activating the PI3K/Akt signaling pathway [42]. Consistent with these results, we showed that knockdown of ANXA3 inhibited the proliferation and migration of microglia, while overexpression of ANXA3 resulted in opposing effects. ANXA3 is a key molecule in membrane ruffling and binds to F-actin in a Ca^2+^-dependent manner [11], suggesting a novel function of ANXA3 in the morphological changes of microglia, which are essential for microglial migration. However, the other functions of ANXA3 in microglia remain to be elucidated.

Our study has some limitations. We only investigated the roles of ANXA3 with regard to the proliferative capacity and migration ability of microglia in vitro; the other functions of microglial ANXA3 in the CNS require further study.

## 5. Conclusions

This study indicates that ANXA3 may be a novel marker for the microglia of adult rats and enriches our understanding of ANXA3 in the CNS from expression patterns to physiological functions, which is vital in the field of microglial ANXA3 research.

## Figures and Tables

**Table 1:** Sequence of primers used for real-time PCR.

Gene	Forward primer (5-3′)	Reverse primer (5-3′)
*CD11b*	CAAGGAGTGTGTTTGCGTGT	AGAAGGCTCGGACAACTGAG
*ANXA3*	CAAATTCACCGAGATCCTGT	TGCTGGAGTGCTGTACGAAA
*Iba1*	TCTGAATGGCAATGGAGATA	GTTGGCTTCTGGTGTTCT
*GAPDH*	GCTCTCTGCTCCTCCCTGTTCTA	TGGTAACCAGGCGTCCGATA

## Data Availability

All data generated or analyzed during this study are included in this published article.

## Abbreviations

ANXA3: Annexin A3
CD11b: Cluster of differentiation 11b
Iba1: Ionized calcium-binding adaptor molecule 1
CNS: Central nervous system
CAMs: CNS-associated macrophages.

## Supplementary Materials

Supplementary Materials The transfection efficiencies of LV-empty, LV-NC, LV-ANXA3, and LV-shANXA3 are provided in the *Supplementary material.*
