# Supplementary material for: Annexin A3 as a Marker Protein for Microglia in the Central Nervous System of Rats
Source: Neural Plast. 2021 Jun 10;2021:5575090. doi: 10.1155/2021/5575090 (PMC8211522; doi:10.1155/2021/5575090)
Supplement: Supplementary Materials — The transfection efficiencies of LV-empty, LV-NC, LV-ANXA3, and LV-shANXA3 are provided in the Supplementary material. [file 5575090.f1.docx]

**Supplementary Information for**

**Annexin A3 as a marker protein for microglia in the central nervous system of rats**

Zengli Zhang^1, 2^, Zhengyiqi Li^1^, Zhi Ma^2^, Meiling Deng^1^, Manyu Xing^1^, Jing Wu^1^, Shasha Jiang^1^, Qiang Wang^1^, Qulian Guo^1^, Wangyuan Zou^1, 3*^

^1^ Department of Anesthesiology, Xiangya Hospital, Central South University, Changsha 410008, China

^2^ Department of Anesthesiology, Center for Brain Science, The First Affiliated Hospital of Xi’an Jiaotong University, Xi’an, China

^3^ National Clinical Research Center for Geriatric Disorders, Xiangya Hospital, Central South University, Changsha 410008, China

**
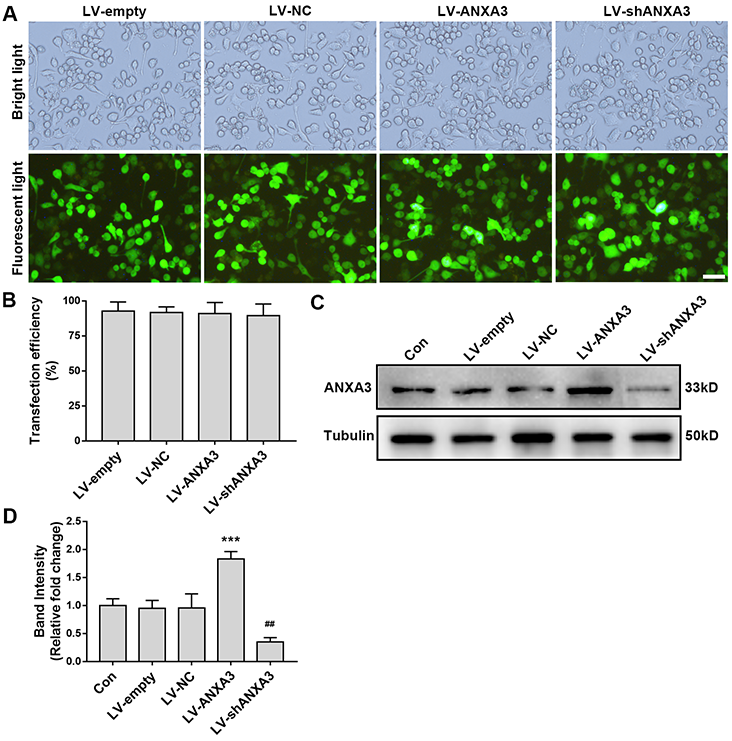
**

**Figure S1. The lentiviral transfection of N9 cells**

(**A-B**) The N9 microglial cells that were successfully transfected (LV-ANXA3 and LV-shANXA3) were photographed under bright-field (left) and fluorescent (right) conditions. The transfection efficiency of lentivirus was the number of successfully transfected cells/the number of total cells in the same field of view. Scale bar =20 μm. (**C-D**) Up- and downregulation of ANXA3 protein expression was identified by Western blotting. The panel shows ANXA3 and tubulin protein bands. The data are expressed as the means ± SD and were analyzed by one-way ANOVA with Tukey’s post hoc test. ****p* <0.001 compared with the LV-empty group. ^##^*p* <0.01 compared with the LV-NC group. The data were pooled from six independent experiments.
